# Targeted Next-Generation Sequencing Identified Compound Heterozygous Mutations in *MYO15A* as the Probable Cause of Nonsyndromic Deafness in a Chinese Han Family

**DOI:** 10.1155/2020/6350479

**Published:** 2020-06-15

**Authors:** Longhao Wang, Lin Zhao, Hu Peng, Jun Xu, Yun Lin, Tao Yang, Hao Wu

**Affiliations:** ^1^Department of Otorhinolaryngology-Head and Neck Surgery, Xinhua Hospital, Shanghai Jiao Tong University School of Medicine, Shanghai 200092, China; ^2^Ear Institute, Shanghai Jiao Tong University School of Medicine, Shanghai 200125, China; ^3^Shanghai Key Laboratory of Translational Medicine on Ear and Nose Diseases, Shanghai 200125, China; ^4^Department of Health Management Center, Shanghai Eastern Hepatobiliary Surgery Hospital, Shanghai 200438, China; ^5^Department of Otorhinolaryngology-Head and Neck Surgery, Changzheng Hospital, Second Military Medical University, Shanghai 200003, China; ^6^Department of Otorhinolaryngology-Head and Neck Surgery, Ninth People's Hospital, Shanghai Jiao Tong University School of Medicine, Shanghai 200011, China

## Abstract

Hearing loss is a highly heterogeneous disorder, with more than 60% of congenital cases caused by genetic factors. This study is aimed at identifying the genetic cause of congenital hearing loss in a Chinese Han family. Auditory evaluation before and after cochlear implantation and targeted next-generation sequencing of 140 deafness-related genes were performed for the deaf proband. Compound heterozygous mutations c.3658_3662del (p. E1221Wfs∗23) and c.6177+1G>T were identified in *MYO15A* as the only candidate pathogenic mutations cosegregated with the hearing loss in this family. These two variants were absent in 200 normal-hearing Chinese Hans and were classified as likely pathogenic and pathogenic, respectively, based on the ACMG guideline. Our study further expanded the mutation spectrum of *MYO15A* as the c.3658_3662del mutation is novel and confirmed that deaf patients with recessive *MYO15A* mutations have a good outcome for cochlear implantation.

## 1. Introduction

Approximately one in every 1000 newborns is affected by congenital hearing loss, and genetic factors account for more than 60% of them [1]. To date, more than 100 deafness-causative genes have been found. Among them, autosomal recessive nonsyndromic hearing loss (ARNSHL) accounts for up to 80% of nonsyndromic hearing loss [2], with more than 70 causative genes being identified (http://hereditaryhearingloss.org/).

Stereocilia is critical for the development and function of cochlear hair cells (HCs) [3–5]. The *MYO15A* gene contains 66 coding exons [6], which encode an unconventional myosin (myosin XVA) expressed at the tips of stereocilia in the cochlear HCs. Myosin XVA is essential for the mechanotransduction function of cochlear HCs. Myosin XVA interacts with the PDZ domain of whirlin and then delivers whirlin to the tips of stereocilia [7]. Myosin XVA-deficient mouse (shaker-2) shows abnormally short stereocilia bundles and diminished staircase [8–10]. In humans, mutations in *MYO15A* have been found to lead to recessive nonsyndromic deafness DFNB3 [11]. The prevalence of *MYO15A* mutations varies among different ethnic populations (3%-6.7%) and appears to be the third or fourth most frequent causes of ARNSHL [12–15].

Here, we report a nonconsanguineous Chinese Han family with profound ARNSHL, in which compound heterozygous mutations in *MYO15A* were identified as the probable cause of the deafness.

## 2. Materials and Methods

### 2.1. Subjects

**Figure 1:**
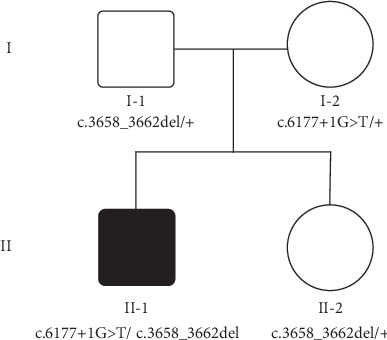
Pedigree and genotype of the Chinese Han family with *MYO15A* mutations.

**Figure 2:**
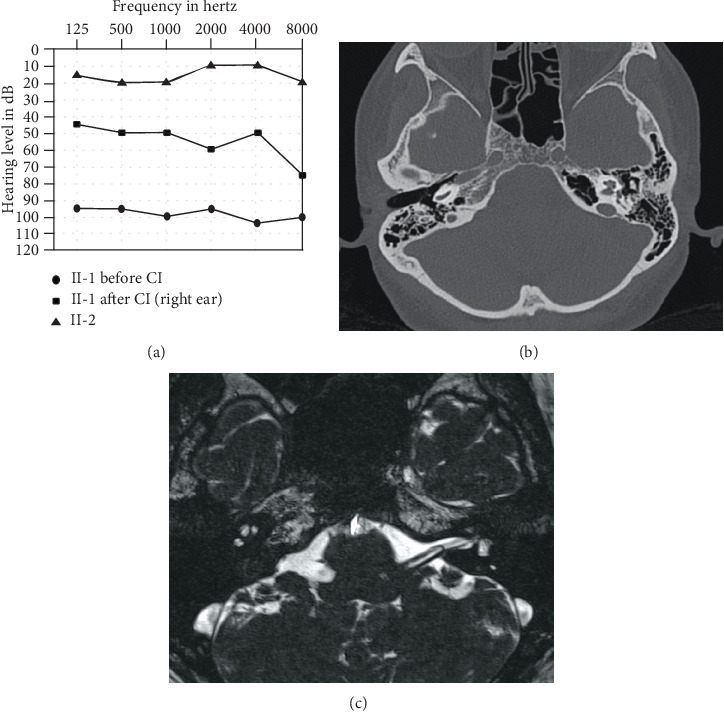
(a) Audiogram of the proband (II-1) before and after cochlear implantation and that of his unaffected sister (II-2). (b) Temporal bone Computerized Tomography (CT) scan of the proband (II-1). (c) Cranial Magnetic Resonance Imaging (MRI) of the proband (II-1).

A Chinese Han recessive deafness family (Figure 1) was enrolled in this study. All family members underwent clinical evaluation in the Department of Otolaryngology-Head and Neck Surgery, Xinhua Hospital, Shanghai Jiao Tong University School of Medicine. The evaluation included a detailed clinical interview and physical examination. As shown in Figure 2(a), the proband had bilateral profound deafness. This study was approved by the ethnic committee of Xinhua Hospital. Written informed consent was obtained for each participant.

### 2.2. Audiometric Evaluation

Audiometric assessments included otoscopic examination, pure tone audiometry (PTA), auditory brainstem response (ABR), and multiple steady-state responses (ASSR). Hearing level was assessed at 250, 500, 1000, 2000, 4000, and 8000 Hz. The hearing threshold was defined as the average of both sides. Inner-ear malformation and dysplasia of the auditory nerve related to the hearing loss were excluded by temporal bone Computerized Tomography (CT) scan and cranial Magnetic Resonance Imaging (MRI).

### 2.3. Mutation Identification

Blood samples were collected into an EDTA anticoagulant tube by venipuncture of the cubital vein. Extraction of genomic DNA was performed using a blood DNA extraction kit (QIAamp DNA Blood Mini Kit, Qiagen, Shanghai). As the first step, mutations in common deafness genes *GJB2*, *SLC26A4*, and *MT-RNR1* were excluded by Sanger sequencing. Targeted next-generation sequencing was then performed in the proband as previously reported [16]. A total of 140 known deafness-related genes were captured by a customized capture assay (MyGenostics, Beijing, China) (Supplementary Table 1). The targeted region included exon, splicing sites, and flanking intron region. Then, potentially candidate variants such as missense, nonsense, and indel variants and the splice site were screened for quality, and variants with minor allele frequencies (MAFs) below 0.005 were further studied using public databases including dbSNP, 1000 Genomes Project, and Exome Aggregation Consortium (EXAC) and in-house data from 200 ethnically matched normal-hearing controls. Intrafamilial segregation of the candidate mutations was examined by Sanger sequencing. The potential pathogenic effects of the candidate mutations were predicted by computational tools including PolyPhen-2, SIFT, and PROVEAN and classified following the American College of Medical Genetics and Genomics (ACMG) guidelines for the interpretation of sequence variants in 2015 [17]. Human Splicing Finder (HSF) (http://www.umd.be/HSF3/) was used to calculate the consensus values of potential splice sites.

## 3. Results

### 3.1. Clinical Characterization

The proband was a 14-year-old male from Zhejiang Province, China. He had congenital, bilateral, profound hearing impairment with a threshold above 95 dBHL as revealed by the PTA (Figure 2(a)) and ABR tests. Hearing levels of this patient and his sister were normal. Otoacoustic emissions were absent for both ears. Temporal CT and cranial MRI showed no abnormalities (Figures 2(b) and 2(c)). No vestibular dysfunction was complained. No apparent syndromic features were found in the physical examination. The proband received unilateral cochlear implantation (Nucleus 5, Cochlear Corporation, Australia) through a typical round window route uneventfully at 12 years old. Hearing was markedly improved after cochlear implantation (Figure 2(a)).

### 3.2. Mutation Analysis

**Figure 3:**
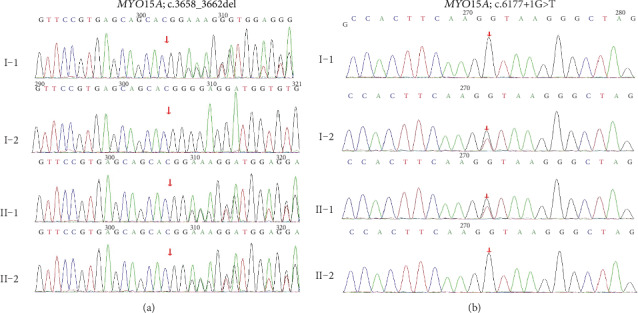
Sanger sequencing results of the c.3658_3662del and c.6177+1G>T mutations in the family members.

By targeted next-generation sequencing of 140 deafness-causative genes in the proband, compound heterozygous mutations c.3658_3662del and c.6177+1>T in *MYO15A* (NM_016239) were identified as the only candidate pathogenic mutations consistent with a presumably autosomal recessive inheritance. The mean depth of sequencing was 364.43X, and 98% of the targeted region was covered with at least 20X. Cosegregation of these two mutations with the hearing phenotype was confirmed within the family members (Figure 3). These two variants were not seen in public databases dbSNP, 1000 Genomes Project, and EXAC and the in-house databases of 200 Chinese Han normal-hearing controls. The frameshifting c.3658_3662del (p.E1221Wfs∗23) mutation is located in exon 3, and it is novel and is predicted to result in a truncated protein after the motor domain (Figure 3). The c.6177+1G>T splice site mutation was previously reported in another Chinese Han family [18] and is predicted to result in an in-frame skipping of exon 26 and a protein product with 17-residue deletion in the first MyTH4 domain. Following the ACMG guideline in 2015 [17], the c.3658_3662del and c.6177+1>T mutations were classified as likely pathogenic (PVS2+PM2) and pathogenic (PVS1+PS1+PM2), respectively.

## 4. Discussion

**Figure 4:**
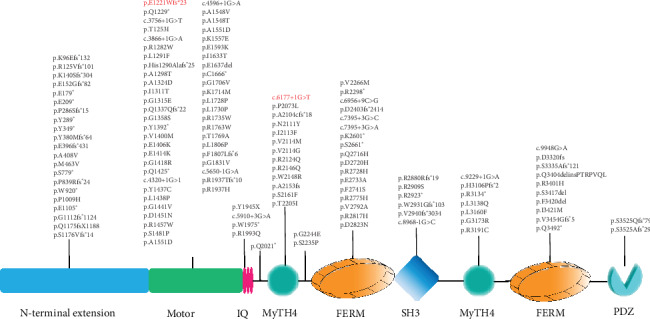
Schematic representation of the reported mutations in *MYO15A* and the corresponding protein structure. Mutations identified in this study were marked in red.

HCs in the cochlea play a critical role in converting mechanical sound waves into neural signals for hearing, and most of the hearing loss induced by gene mutation, noise, different ototoxic drugs, inflammation, or aging is caused by the HC malfunction [19–27]. The association between *MYO15A* mutations and recessive deafness DFNB3 was first discovered by Friedman et al. in Bali, Indonesia [28], in which two missense mutations and one nonsense mutation in *MYO15A*, all in a homozygous state, result in congenital, severe-to-profound hearing loss [11]. To date, more than 100 mutations in *MYO15A* have been reported, mostly reported in consanguineous families from the Middle East [27, 29–35]. In this study, two variants p.E1221Wfs∗23 and c.6177+1G>T in *MYO15A* were identified. Like many previously reported truncating mutations in *MYO15A*, the p.E1221Wfs∗23 variant is predicted to result in a truncated protein product without Motor, IQ, MyTH4, FERM, SH3, and PDZ domains (Figure 4). The c.6177+1G>T variant was previously reported in another Chinese Han family by Chen et al. [18], suggesting that this mutation may be either a founder mutation or a reoccurrent hot spot. This mutation resides in the consensus splice acceptor site adjacent to exon 26 and is predicted to lead to an in-frame exon 26 skipping and a 17-amino acid residue deletion in the first myosin tail homology 4 (MyTH4) domain of myosin XVA. The MyTH4 domain provides a link between actin-based kinesin and the microtubule cytoskeleton. Mutation in this domain can disrupt the protein-protein interaction that is important for mechanotransduction of hearing [7].

Most recessive mutations in *MYO15A* are associated with congenital, severe-to-profound deafness [31, 33, 36], except for mutations affecting the N-terminal domain of MYOXVA which may result in milder hearing loss with residual hearing of low frequency [37]. Both variants identified in our study are located outside of the N-terminal domain, and the associated profound hearing loss is consistent with the genotype-phenotype correlation for DFNB3 deafness. Consistent with the specific role of *MYO15A* in the sensory HCs, the proband in our study had a marked improvement for hearing after cochlear implantation, showing a good prospective outcome for a similar procedure in other DFNB3 patients.

## 5. Conclusion

The p.E1221Wfs∗23 and c.6177+1G>T compound heterozygous mutations in MYO15A are the probable cause of congenital, profound deafness in the Chinese Han family. Patients with recessive mutations in *MYO15A* may markedly benefit from cochlear implantation.

## Data Availability

The data underlying the findings of this study is available upon request.

## Supplementary Materials

Supplementary Materials Supplementary Table 1: the 140 deafness-causative genes for targeted next-generation sequencing.
