# Supplementary material for: Targeted Next-Generation Sequencing Identified Compound Heterozygous Mutations in MYO15A as the Probable Cause of Nonsyndromic Deafness in a Chinese Han Family
Source: Neural Plast. 2020 Jun 15;2020:6350479. doi: 10.1155/2020/6350479 (PMC7313121; doi:10.1155/2020/6350479)
Supplement: Supplementary Materials — Supplementary Table 1: the 140 deafness-causative genes for targeted next-generation sequencing. [file 6350479.f1.docx]

**Supplementary Table S1.** 140 genes targeted for the next-generation sequencing

| [*ACTG1*](http://www.ncbi.nlm.nih.gov/omim/102560)*^N^* | *CO1^S^* | *FGF8^S^* | *miR-182^N^* | [*MYH9*](http://www.ncbi.nlm.nih.gov/omim/160775)*^N/S^* | *PROK2^S^* | [*TBC1D24*](http://www.omim.org/entry/613577)*^N/S^* |
| --- | --- | --- | --- | --- | --- | --- |
| *ALX3^S^* | [*COL9A1*](http://www.ncbi.nlm.nih.gov/omim/120210)*^S^* | *FGFR1^S^* | [*HSD17B4*](http://omim.org/entry/601860)*^S^* | *miR-183^S^* | *PROKR2^S^* | [*TCOF1*](http://www.ncbi.nlm.nih.gov/omim/606847)*^S^* |
| [*BSND*](http://www.ncbi.nlm.nih.gov/omim/606412)*^S^* | [*COL9A2*](http://omim.org/entry/120260)*^S^* | *FGFR3^S^* | *IL13^S^* | [*MYO15A*](http://www.ncbi.nlm.nih.gov/omim/602666)*^N^* | [*PRPS1*](http://www.ncbi.nlm.nih.gov/omim/311850)*^S^* | [*TECTA*](http://www.ncbi.nlm.nih.gov/omim/602574)*^N^* |
| *CABP2^N^* | *COMT2^S^* | *FLNA^S^* | [*ILDR1*](http://www.ncbi.nlm.nih.gov/omim/609739)*^N^* | [*MYO3A*](http://www.ncbi.nlm.nih.gov/omim/606808)*^N^* | [*PTPRQ*](http://omim.org/entry/603317)*^N^* | *TIMM8A^S^* |
| [*CCDC50*](http://www.ncbi.nlm.nih.gov/omim/611051)*^N^* | [*CRYM*](http://www.ncbi.nlm.nih.gov/omim/123740)*^N^* | [*FOXI1*](http://www.ncbi.nlm.nih.gov/omim/601093)*^S^* | [*KARS*](http://omim.org/entry/601421)*^N/S^* | [*MYO6*](http://www.ncbi.nlm.nih.gov/omim/600970)*^N^* | [*RDX*](http://www.ncbi.nlm.nih.gov/omim/179410)*^N^* | [*TJP2*](http://www.ncbi.nlm.nih.gov/omim/607709)*^N^* |
| [*CDH23*](http://www.ncbi.nlm.nih.gov/omim/605516)*^N/S^* | [*DFNA5*](http://www.ncbi.nlm.nih.gov/omim/600994)*^N^* | *FREM1^S^* | [*KCNE1*](http://www.ncbi.nlm.nih.gov/omim/176261)*^S^* | [*MYO7A*](http://www.ncbi.nlm.nih.gov/omim/276903)*^N/S^* | *RPGR^S^* | [*TMC1*](http://www.ncbi.nlm.nih.gov/omim/606706)*^N^* |
| [*CEACAM16*](http://www.ncbi.nlm.nih.gov/pubmed/21368133)*^N^* | *DFNB59^N^* | *GATA3^S^* | [*KCNJ10*](http://omim.org/entry/602208)*^N/S^* | [*NDP*](http://www.ncbi.nlm.nih.gov/omim/300658)*^S^* | *SALL1^S^* | [*TMIE*](http://www.ncbi.nlm.nih.gov/omim/607237)*^N^* |
| *CHD7^S^* | *DIABLO^N^* | *GIPC3^N^* | [*KCNQ1*](http://www.ncbi.nlm.nih.gov/omim/607542)*^S^* | *NF2^N^* | *SALL4^S^* | [*TMPRSS3*](http://www.ncbi.nlm.nih.gov/omim/605511)*^N^* |
| [*CIB2*](http://omim.org/entry/605564)*^N^* | [*DIAPH1*](http://www.ncbi.nlm.nih.gov/omim/602121)*^N^* | [*GJB2*](http://www.ncbi.nlm.nih.gov/omim/121011)*^N/S^* | [*KCNQ4*](http://www.ncbi.nlm.nih.gov/omim/603537)*^N^* | [*OTOA*](http://www.ncbi.nlm.nih.gov/omim/607038)*^N^* | *SEC23A^S^* | [*TNC*](http://www.omim.org/entry/187380)*^N^* |
| [*CLDN14*](http://www.ncbi.nlm.nih.gov/omim/605608)*^N^* | *DIAPH3^N^* | *GJB3^N/S^* | *KRT9^S^* | [*OTOF*](http://www.ncbi.nlm.nih.gov/omim/603681)*^N^* | [*SEMA3E*](http://www.omim.org/entry/608166)*^S^* | [*TPRN*](http://www.ncbi.nlm.nih.gov/omim/613354)*^N^* |
| *CLPP^S^* | [*DSPP*](http://www.ncbi.nlm.nih.gov/omim/125485)*^S^* | [*GJB6*](http://www.ncbi.nlm.nih.gov/omim/604418)*^N^* | *LAMA3^S^* | [*P2RX2*](http://www.omim.org/entry/600844)*^N^* | [*SERPINB6*](http://omim.org/entry/173321)*^N^* | [*TRIOBP*](http://www.ncbi.nlm.nih.gov/omim/609761)*^N^* |
| [*CLRN1*](http://www.ncbi.nlm.nih.gov/omim/606397)*^S^* | *ECM1^U^* | *GPR98^S^* | [*LARS2*](http://omim.org/entry/604544)*^S^* | *PABPN1^S^* | [*SIX1*](http://www.omim.org/entry/601205)*^N/S^* | *TRMU^S^* |
| [*COCH*](http://www.ncbi.nlm.nih.gov/omim/603196)*^N^* | [*EDN3*](http://www.ncbi.nlm.nih.gov/omim/131242)*^S^* | [*GPSM2*](http://omim.org/entry/609245)*^S^* | [*LHFPL5*](http://www.ncbi.nlm.nih.gov/omim/609427)*^N^* | [*PAX3*](http://www.ncbi.nlm.nih.gov/omim/606597)*^S^* | [*SIX5*](http://www.ncbi.nlm.nih.gov/omim/600963)*^S^* | [*TSPEAR*](http://www.omim.org/entry/612920)*^N^* |
| [*COL11A1*](http://www.ncbi.nlm.nih.gov/omim/120280)*^N/S^* | [*EDNRB*](http://www.ncbi.nlm.nih.gov/omim/131244)*^S^* | *GRHL2^N^* | [*LOXHD1*](http://www.ncbi.nlm.nih.gov/omim/613072)*^N^* | [*PCDH15*](http://www.ncbi.nlm.nih.gov/omim/605514)*^N^/^S^* | [*SLC17A8*](http://omim.org/entry/607557)*^N^* | [*USH1C*](http://www.ncbi.nlm.nih.gov/omim/605242)*^N/S^* |
| [*COL11A2*](http://www.ncbi.nlm.nih.gov/omim/120290)*^N/S^* | [*ELMOD3*](http://www.omim.org/entry/615427)*^N^* | [*GRXCR1*](http://www.ncbi.nlm.nih.gov/omim/613283)*^N^* | *LRTOMT^N^* | [*PDZD7*](http://www.ncbi.nlm.nih.gov/omim/612971)*^N^* | [*SLC26A4*](http://www.ncbi.nlm.nih.gov/omim/605646)*^N/S^* | *USH1G^S^* |
| [*COL2A1*](http://www.ncbi.nlm.nih.gov/omim/120140)*^S^* | [*ESPN*](http://www.ncbi.nlm.nih.gov/omim/606351)*^N^* | *HARS^S^* | [*MARVELD2*](http://www.ncbi.nlm.nih.gov/omim/610572)*^N^* | [*PNPT1*](http://omim.org/entry/610316?search=PNPT1&highlight=pnpt1)*^N^* | [*SLC26A5*](http://www.ncbi.nlm.nih.gov/omim/604943)*^N^* | [*USH2A*](http://www.ncbi.nlm.nih.gov/omim/276901)*^S^* |
| [*COL4A3*](http://www.ncbi.nlm.nih.gov/omim/120070)*^S^* | [*ESRRB*](http://www.ncbi.nlm.nih.gov/omim/602167)*^N^* | [*HARS2*](http://omim.org/entry/600783)*^S^* | *MIR96^N^* | [*POLR1C*](http://www.omim.org/entry/610060)*^S^* | [*SMPX*](http://omim.org/entry/300226)*^N/S^* | [*WFS1*](http://www.ncbi.nlm.nih.gov/omim/606201)*^N/S^* |
| [*COL4A4*](http://www.ncbi.nlm.nih.gov/omim/120131)*^S^* | [*EYA1*](http://www.ncbi.nlm.nih.gov/omim/601653)*^S^* | [*HGF*](http://www.ncbi.nlm.nih.gov/omim/142409)*^N^* | [*MITF*](http://www.ncbi.nlm.nih.gov/omim/156845)*^S^* | [*POLR1D*](http://www.omim.org/entry/613715)*^S^* | [*SNAI2*](http://www.ncbi.nlm.nih.gov/omim/602150)*^S^* | *WHRN^N/S^* |
| [*COL4A5*](http://www.ncbi.nlm.nih.gov/omim/303630)*^S^* | [*EYA4*](http://www.ncbi.nlm.nih.gov/omim/603550)*^N/S^* | *HMX1^S^* | [*MSRB3*](http://www.ncbi.nlm.nih.gov/omim/613719)*^N^* | [*POU3F4*](http://www.ncbi.nlm.nih.gov/omim/300039)*^N^* | [*SOX10*](http://www.ncbi.nlm.nih.gov/omim/602229)*^S^* | *MT-RNR1^N^* |
| [*COL4A6*](http://www.omim.org/entry/303631)*^N/S^* | *FGF3^S^* | *HOXA2^S^* | [*MYH14*](http://www.ncbi.nlm.nih.gov/omim/608568)*^N/S^* | [*POU4F3*](http://www.ncbi.nlm.nih.gov/omim/602460)*^N^* | [*STRC*](http://www.ncbi.nlm.nih.gov/omim/606440)*^N^* | *MT-TS1^S^* |

*N: nonsyndromic hearing loss; S: syndromic hearing loss.
